# Interventional Left Atrial Appendage Closure Affects the Metabolism of Acylcarnitines

**DOI:** 10.3390/ijms19020500

**Published:** 2018-02-07

**Authors:** Christian Fastner, Michael Behnes, Benjamin Sartorius, Annika Wenke, Siegfried Lang, Gökhan Yücel, Katherine Sattler, Jonas Rusnak, Ahmad Saleh, Christian Barth, Kambis Mashayekhi, Ursula Hoffmann, Martin Borggrefe, Ibrahim Akin

**Affiliations:** 1 First Department of Medicine, University Medical Center Mannheim, Faculty of Medicine Mannheim, University of Heidelberg, 68167 Mannheim, Germany; christian.fastner@umm.de (C.F.); sartorius@stud.uni-heidelberg.de (B.S.); wenke@stud.uni-heidelberg.de (A.W.); siegfried.lang@umm.de (S.L.); goekhan.yuecel@umm.de (G.Y.); katherine.sattler@umm.de (K.S.); jonas.rusnak@umm.de (J.R.); ahmad.saleh@umm.de (A.S.); christian.barth@umm.de (C.B.); ursula.hoffmann@umm.de (U.H.); martin.borggrefe@umm.de (M.B.); ibrahim.akin@umm.de (I.A.); 2 Division of Cardiology and Angiology II, University Heart Center Freiburg-Bad Krozingen, 79189 Bad Krozingen, Germany; kambis.mashayekhi@universitaets-herzzentrum.de

**Keywords:** atrial fibrillation, left atrial appendage, left atrial appendage closure, metabolomics, acylcarnitines

## Abstract

Background: Left atrial appendage closure (LAAC) represents the interventional alternative to oral anticoagulation for stroke prevention in atrial fibrillation (AF). The metabolism of acylcarnitines was shown to affect cardiovascular diseases. This study evaluates the influence of successful LAAC on the metabolism of acylcarnitines. Methods: Patients undergoing successful LAAC were enrolled prospectively. Peripheral blood samples for metabolomics measurements were collected immediately before (i.e., index) and six months after LAAC (i.e., mid-term). A targeted metabolomics analysis based on electrospray ionization–liquid chromatography–mass spectrometry (ESI–LC–MS/MS) and MS/MS measurements was performed. Results: 44 patients with non-valvular AF (median CHA_2_DS_2_-VASc score 4, median HAS-BLED score 4) and successful LAAC were included. Significant changes in acylcarnitine levels were found in the total cohort, which were mainly attributed to patients with impaired left ventricular and renal function, elevated amino-terminal pro-brain natriuretic peptide (NT-proBNP) and diabetes mellitus. Adjusted multivariable regression models revealed significant changes of five metabolites over mid-term follow-up: C2, C14:1, C16, and C18:1 decreased significantly (each *p* < 0.05); short-chain C5 acylcarnitine plasma levels increased significantly (*p* < 0.05). Conclusion: This study demonstrates that successful LAAC affects the metabolism of acylcarnitines at mid-term follow-up. Clinical Trial Registration: ClinicalTrials.gov Identifier: NCT02985463.

## 1. Introduction

The catheter-based left atrial appendage closure (LAAC) is an emerging alternative to oral anticoagulation (OAC) for the prevention of stroke and systemic embolization in non-valvular atrial fibrillation (AF) patients prone to a high bleeding risk under long-term OAC [1,2].

A large effort has been made to investigate the efficacy and safety of LAAC. For the WATCHMAN™ device (Boston Scientific, Natick, MA, USA), data of a randomized controlled trial exist [3]. Non-inferiority and superiority compared to OAC for preventing the combined outcome of stroke, systemic embolization, and cardiovascular death could be shown. Moreover, the superiority for cardiovascular and all-cause mortality was demonstrated [3].

From the perspective of an interventional cardiologist, the LAA has become of growing scientific interest. It is not only the main source of thromboembolism during non-valvular AF, but also reveals relevant impact on neuro-humoral hemostasis [4]. It was shown recently that LAAC might alter hormone homeostasis, as reflected by decreasing levels of atrial natriuretic peptide at both short- and mid-term follow-up [5,6,7].

The metabolome may reflect relevant aspects for the course and severity of predefined cardiac diseases [8,9]. Specific metabolites can be measured from peripheral blood and, thus, are easily accessible for diagnostic evaluation [9]. Additionally, cardiac interventions were shown to affect peripheral levels of the tricarboxylic acid (TCA) derivate [10,11]. In this context, metabolomic profiling appears also suitable to investigate an intervention´s impact, such as that of LAAC, on cardiac metabolism [7,12].

As a part of the complex metabolome, acylcarnitines not only provide medium-chain (MC) and long-chain (LC) fatty acids to β-oxidation in the mitochondrial matrix, a very relevant source of energy for the heart muscle, but also appear to directly interfere with some cellular membrane-associated systems also including ion channels [13]. Elevated serum levels of acylcarnitines were shown to be linked to an increased cardiovascular morbidity, especially in the case of coronary artery disease and heart failure [14,15,16]. Therefore, acylcarnitines are known for their important role in human body’s metabolism especially concerning energy supply in highly active tissues [17]. However, the influence of LAAC—an energy-demanding cardiac intervention within the LAA—on the metabolism of acylcarnitines has never been evaluated. 

Accordingly, this study primarily aims to investigate whether LAAC affects the metabolism of acylcarnitines at mid-term follow-up, as analyzed with a targeted, quantitative metabolomics approach.

## 2. Results

### 2.1. Baseline Characteristics and Procedures’ Indications

The samples of 44 patients were analyzed. Baseline characteristics, as well as the percentages of adverse events and reasons for rehospitalization between the intervention (T1) and mid-term follow-up (T2) are displayed in Table 1. The patients presented as a collective with a relevantly increased cardiovascular risk profile (diabetes mellitus in 36.7%) and both a high stroke (median CHA_2_DS_2_-VASc score 4) and a high bleeding risk (median HAS-BLED score 4). The main indication for LAAC device implantation was a prior bleeding event (77.3%).

Based on a thorough clinical assessment, there were no relevant changes in the overall clinical status over mid-term follow-up, as reflected by a stable course regarding nutritional and smoking status, pharmacotherapy, heart failure symptoms, anemia, renal function, lipid status, and inflammation (each *p* > 0.05 between T1 and T2; Table 2). No significant differences were found regarding the impairment of left ventricular (LV) function over time (each *p* > 0.05).

**Figure 1:**
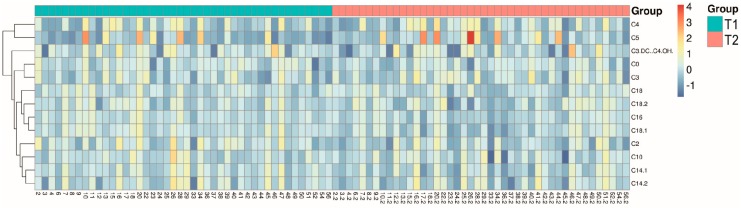
Hierarchical cluster analysis giving an overview of differences in metabolites between T1 and T2.

A hierarchical cluster analysis is shown as Figure 1, which allows an overview of the differences between T1 and T2 for certain metabolites.

### 2.2. Numerical Non-Adjusted Changes of Acylcarnitine Levels

**Figure 2:**
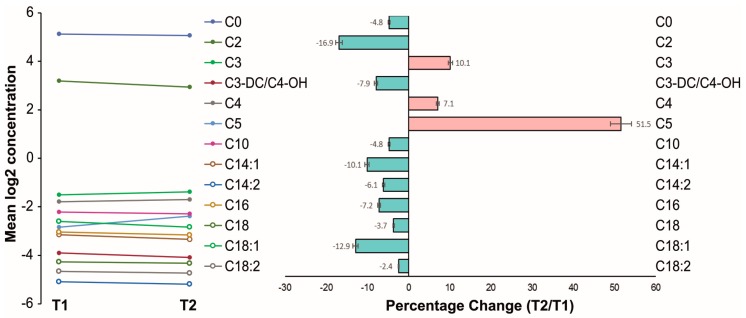
Changes of acylcarnitines before and after left atrial appendage closure over mid-term follow-up. (**Left**) Mean logarithmic changes of carnitine and acylcarnitine plasma levels for each analyzed metabolite; (**right**) percentage change.

Figure 2 shows the logarithmic changes of the carnitine and acylcarnitine plasma levels for each analyzed metabolite. Numerically, the percentage change (Figure 2) of all MC and LC acylcarnitine levels was negative over mid-term follow-up. Also, the mean plasma level of carnitine itself decreased numerically. In contrast, the percentage change of most short-chain (SC) acylcarnitine levels was positive. Noteworthy, significant overall changes were found for C2, C5, C14:1, and C18:1 over mid-term follow-up (*p* < 0.05; Table 3).

### 2.3. Subgroup Analyses

In a second step, relevant subgroups potentially influencing the acylcarnitine metabolism were adjusted separately in order to reveal their influence in the present unselected study cohort. Particularly worthy of note in respect of the subgroup analyses (Supplemental Tables S1–S4) is that neither gender, age, nor body mass index (BMI) significantly influenced the changes in acylcarnitine plasma levels. In contrast, the plasma levels of C18 and C3 revealed a significantly different change in patients with a normal left ventricular ejection fraction (LVEF) compared to those with a reduced LVEF. The changes in C5 and C16 plasma levels were significantly different in patients with a higher amino-terminal pro-brain natriuretic peptide (NT-proBNP) value compared to those with a lower NT-proBNP value. In the case of impaired renal function, reflected by an increased creatinine value (i.e., >1.2 mg/dL), the changes of the LC acylcarnitines C18:1, C14:1, and C16 revealed significant differences compared to those in patients with normal renal function. In patients with diabetes mellitus, carnitine, as well as C18:1, C18:2, and C16 plasma level changes were significantly different from those of patients without diabetes mellitus.

### 2.4. Adjusted Multivariable Regression Model

By adjusting for all confounding factors based on subgroup analyses, a multivariable linear regression model was evaluated. In this model, the initial significant metabolites and C16 revealed significant changes after LAAC procedure, as seen in Table 4.

**Figure 3:**
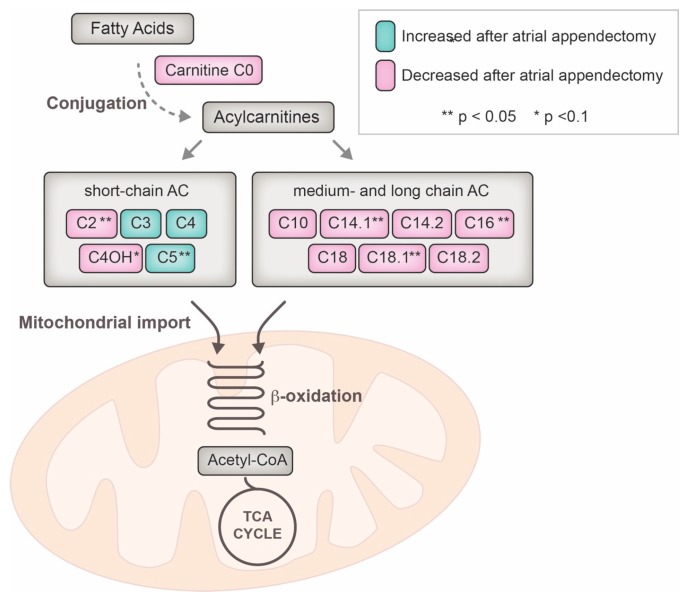
Metabolomic pathway of acylcarnitine utilization and influence of the left atrial appendage closure over the mid-term follow-up. ** indicate statistical significance after regression analysis (*p* < 0.05), * indicates a statistical trend (*p* < 0.1); AC = acylcarnitine, CoA = co-enzyme A, TCA = tricarboxylic acid.

Figure 3 finally summarizes the pathway changes of acylcarnitine plasma levels clustered by the length of their conjugated fatty acid and their link to the mitochondrial tricarboxylic acid cycle.

## 3. Discussion

The present observational and hypothesis-generating study investigates whether successful interventional LAAC may affect the metabolism of acylcarnitines. It was demonstrated that LAAC was associated with statistically significant changes of certain metabolites of the acylcarnitine pathway at mid-term follow-up.

The self-expanding implanted LAAC device exerts an eccentric and permanent stretch at the LAA landing zone. This is due to the recommended device compression of at least 20% for optimal placement [18]. Until neo-endothelialization is completed, a process that has been reported to vary during mid-term follow-up or even afterwards [19,20,21], the blood still circulates through the meshes of the device. However, freedom from thromboembolism out of the LAA is warranted, unless all lobes are covered, and no major peri-device leak of more than 5 mm is detected [20,21,22]. Within the occluded LAA, thrombosis occurs over time but may still be incomplete at mid-term follow-up, as assessed by our own computed tomography (CT) database [21]. A complete obliteration after LAAC at mid-term follow-up may not occur and LAAC does not necessarily correspond to the LAA’s complete exclusion from systemic circulation [20,21]. Moreover, the local wall stress might be even aggravated within the remaining non-thrombosed parts of the LAA, as well as because of ongoing contraction of the LAA within the cardiac cycle. Based on the expounded evidence, it might be hypothesized that these mechanisms trigger an altered energy demand within the myocytes of the LAA, and this might in turn be reflected by the presented decrease of the acylcarnitine metabolism.

To understand why the circulating acylcarnitine levels may be changed after LAAC, it is important to know that these intermediates of the β-oxidation pathway are esterified by the carnitine palmitoytransferase (CPT) 1 in the outer membrane of the mitochondria, out of a fatty acyl-CoA and a carnitine molecule. Then, they are transported across the membrane by the carnitine–acylcarnitine carrier protein (CAC) and reseparated by the C(O-)PT 2 in the inner membrane [13]. This mechanism can act in a duplex mode: under conditions yielding high intramitochondrial acylcarnitine levels, these molecules can also be transported out of the mitochondria and out of the cell [13,23]. Translated to a working model, the changes in acylcarnitine plasma levels might, therefore, be based (A) on modifications of the transmembrane transporting system and (B) on the intramitochondrial acyl-CoA levels which are connected to the velocity of β-oxidation. This study shows evidence that LAAC might influence these pathways.

Moreover, alterations of acylcarnitines were shown to be associated with an adverse cardiovascular outcome in different disease groups, including heart failure, coronary artery disease, and hemodialysis [14,15,16,24]. Increased levels were associated with adverse events or mortality; decreased values were shown to be potentially protective [15]. These prognostic effects were explained by cellular stress, mitochondrial alterations, or the increase of cellular inflammation [13,25]. Noteworthy, these effects were more particularly attributed to specific acylcarnitines, for instance, to LC acylcarnitines (C16 and C18:1) [14]. Hence, LC acylcarnitine levels have been identified as therapeutic targets in heart failure patients [17], and reduced plasmatic LC acylcarnitine levels after LAAC (C14:1, C16 and C18:1 in the present study) might, therefore, directly contribute to lower cellular inflammation processes [13,25]. 

There was a considerable part of patients with diabetes mellitus undergoing the LAAC procedure (36.7%). In a subgroup analysis, the LC acylcarnitine levels in these patients were not as extensively modified by the procedure as in patients without a diabetic disorder. This finding might be attributed to the fact that, in patients with insulin resistance, the LC acylcarnitine levels are elevated by nature [25,26,27] and, consequently, cannot be affected by the LAAC procedure to the same extent. On the other hand, the patients with an impaired renal function, characterized by a creatinine level >1.2 mg/dL at the time of hospital admission, revealed a higher decrease of certain LC acylcarnitine levels after the procedure. It might be speculated whether this decrease of LC acylcarnitines after LAAC, which was observed in this study, might influence the cardiovascular outcome and prognosis of patients with impaired renal function [24] on the basis of a reduced inflammation and cellular stress reduction [13].

The assessment of metabolites is very complex and is usually influenced by nutritional and functional capacities. However, no relevant clinical changes in both nutritional and functional status were found in the present study cohort over mid-term follow-up. Several clinical and biochemical markers including smoking status, pharmacotherapy, anemia, renal function, nutritional status, inflammation, and heart failure did not differ significantly in the follow-up.

### Study Limitations

The present study represents a prospective, non-randomized observational study revealing a hypothesis-generating character and demonstrating only associations between LAAC and alterations of acylcarnitine metabolism. However, the baseline characteristics in the present cohort reflect an increased cardiovascular risk, which is of practical clinical relevance, considering the recent large multicenter registries on LAAC [28]. This single cohort was not replicated and not compared to non-intervened control patients, which might bias the interpretation of the results. Because of the all-comers fashion of enrolment, a certain bias in patients’ selection may have occurred, which was adjusted through our step-wise statistical adjustment including the present subgroups of this population. By the targeted metabolomics approach only a limited number of acylcarnitines was selected based on the assumption of their practical relevance reflected by the existing literature of acylcarnitines in cardiac diseases [14,15,16,24]. The alterations of metabolites in peripheral blood samples after LAAC might only reflect an indirect association without a definitive cause–effect relation. Moreover, the impact of the registered changes in acylcarnitines on patients’ outcome was beyond the focus of this study. These issues should, therefore, be addressed by further prospective randomized studies based on the hypotheses being generated by this study.

## 4. Materials and Methods

The “Left Atrial Appendage Occlusion and Biomarker Evaluation” (LABEL) study (ClinicalTrials.gov Identifier: NCT02985463) is a single-center, prospective, observational non-randomized study including patients being eligible for percutaneous LAAC.

### 4.1. Sample Collection

The peripheral blood samples of 44 consecutive patients undergoing LAAC procedure by either the WATCHMAN™ device or the Amplatzer™ Amulet™ (St. Jude Medical, St. Paul, MN, USA) in our center between 2014 and 2016 were collected within 24 hours prior to cardiac intervention (T1) in serum and ethylenediaminetetra-acetic acid (EDTA) tubes (Sarstedt, Nümbrecht, Germany). Inclusion and exclusion criteria for the LAAC procedure, as well as the conduction of the procedure have been previously reported [6], and were in line with European guideline recommendations on atrial fibrillation [1]. In the context of the routine clinical follow-up six months after the procedure (i.e., mid-term, T2), a second peripheral blood sample was collected from all patients with an initial successful implantation (i.e., stable device position and peri-device leak ≤ 5 mm confirmed by transesophageal echocardiography and cardiac CT). Immediately after the collection, all blood samples from T1 and T2 were stored at 4 °C until further processing. Each sample was centrifuged at 2500× *g* for 10 min at 20 °C, and the aliquoted samples were cooled down with liquid nitrogen before being stored at −80 °C until analysis. The whole processing took place within two hours after blood extraction. A written informed consent was obtained from all participants. The study was carried out according to the principles of the Helsinki Declaration and was approved by the local medical ethics committee II of the Faculty of Medicine Mannheim, University of Heidelberg, Mannheim, Germany (local ethical approval number: 2014-402M-MA-§ 23b MPG, date of approval: 3 November 2014).

### 4.2. Characterization of the Study Population and Changes over the Mid-Term Follow-Up

At T1 and T2, the following characteristics were thoroughly assessed in-hospital and in collaboration with general practitioners on the basis of clinical judgment: relevant changes of body weight or dietary habits (i.e., nutritional status), clinical signs of heart failure (e.g., dyspnea, edema), renal failure (e.g., fatigue, pruritus, edema), (pre)diabetes mellitus (e.g., fatigue, polydipsia, polyuria) and local or systemic inflammation. In addition, BMI, cholesterol, low- and high-density lipoproteins, triglycerides, LV function, NT-proBNP, creatinine level and glomerular filtration rate calculated by the Modification of Diet in Renal Disease (MDRD) formula, average blood glucose level, C reactive protein (CRP), and lactate dehydrogenase (LDH) were measured and assessed at baseline. These parameters were re-assessed occasionally during follow-up on the basis of clinical judgment according to the patients’ individual history or clinical deterioration.

### 4.3. Metabolite Analyses

A targeted metabolomics approach based on electrospray ionization–liquid chromatography–mass spectrometry (ESI–LC–MS/MS) and MS/MS measurements was performed using the AbsoluteIDQ™ p180 Kit (Biocrates Life Sciences, Innsbruck, Austria). The assay allows the simultaneous quantification of a total of 188 metabolites from 10 µL plasma samples. This study focuses on the quantification of carnitine, as well as of the C2, C3, C3-DC/C4-OH, C4, C5, C10, C14:1, C14:2, C16, C18, C18:1, and C18:2 acylcarnitines. The analyses were carried out on a 4000 QTRAP^®^ System (Sciex Deutschland, Darmstadt, Germany) and a Thermo Scientific™ TSQ™ (ThermoFisher Scientific, Waltham, MA, USA). For the evaluation of the metabolites’ concentrations, internal standards served as a reference. The MetIDQ™ (Biocrates Life Sciences, Innsbruck, Austria) software was used for the processing and the technical validation of the metabolite data.

### 4.4. Outcome Measures

The primary outcome measure was the net change in the plasma levels of the different acylcarnitines prior to and after the LAAC procedure. Subgroup analyses were performed for gender, age, BMI, LVEF, NT-proBNP and creatinine levels, as well as for the presence of diabetes mellitus. These parameters are known to influence the metabolism of patients, and the present study cohort was, therefore, adjusted for these factors, as assessed at baseline.

### 4.5. Statistics

Prior to the first patient’s inclusion, a power calculation was performed: based on a false discovery rate (FDR) < 0.05 (complying with the alpha level), a power of 1.00 could be achieved in at least 40 patients. To exclude metabolites whose concentration values were below the limit of detection (LOD), a general cleaning of the dataset based on an 80% rule was performed. The remaining values below the LOD in the dataset were then imputed by applying a logspline imputation method, and the resulting dataset was log2 transformed [29,30]. Principal Component Analysis (PCA), Partial Least Squares–Discrimination Analysis (PLS–DA), and Hierarchical Cluster Analysis (HCA) were used as supervised and unsupervised multivariate approaches [31]. To compare the differences, the scaled data were subjected to a dependent Student’s *t*-test, the Fisher’s exact test or to the repeated measures Analysis of Variance (rANOVA). To control the FDR during multiple comparisons, an adjusted *p* value (Benjamini-Hochberg correction) was additionally calculated [32]. The percentage change from T1 to T2 was calculated by the equation $\frac{\left[mean\;concentration\;at\;T2\right]-\left[mean\;concentration\;at\;T1\right]}{\left[mean\;concentration\;at\;T1\right]}*100$. A regression analysis based on a linear mixed-effect model was applied for the evaluation of significant metabolites dependent on all seven subgroups (gender, age, BMI, LVEF, NT-proBNP and creatinine levels, as well as the presence of diabetes mellitus). The baseline characteristics are presented as medians with interquartile ranges (25th and 75th percentiles) or as frequencies with percentages. To compare the scaled data over the mid-term follow-up, a dependent student’s t-test was applied. The categorical variables were compared using a Fisher’s exact test. The statistical analysis was performed using RStudio (RStudio, Boston, MA, USA) and SPSS Statistics version 22 (IBM, Armonk, NY, USA). *p* < 0.05 was considered significant, a statistical trend was set if *p* < 0.1. All results were based on the available cases.

## 5. Conclusions

This study demonstrated, for the first time, significant alterations of acylcarnitines after successful LAAC, especially of plasma levels of LC acylcarnitines. Future research needs to evaluate their impact on the outcome of this specific subset of interventionally treated patients.

## Figures and Tables

**Table 1:** Baseline characteristics of 44 patients with successful left atrial appendage closure and biomarker evaluation.

Characteristic	Value
**Demographics**	
sex, male *n* (%)	30 (68.2)
age, y (IQR)	78 (75.8–83)
BMI, kg/m^2^ (IQR)	28.1 (24.7–32.7)
**Cardiovascular risk factors**	
arterial hypertension, *n* (%)	42 (95.4)
diabetes mellitus, *n* (%)	16 (36.7)
HbA1c, % (IQR)	7.0 (6.2–7.0)
hypercholesterinemia, *n* (%)	22 (50)
cholesterol, mg/dL (IQR)	143 (124–186)
low-density lipoprotein, mg/dL (IQR)	89 (68–111)
high-density lipoprotein, mg/dL (IQR)	55 (41–62)
triglycerides, mg/dL (IQR)	117 (95–162)
impaired left ventricular function, each *n* (%)	
LVEF 45–54%	4 (9.1)
LVEF 30–44%	4 (9.1)
LVEF < 30%	2 (4.5)
diastolic dysfunction, *n* (%)	11 (25.0%)
combined with impaired left ventricular function, *n* (%)	1 (2.3%)
**Medical history**	
atrial fibrillation, each *n* (%)	
Paroxysmal	24 (54.5)
Persistent	6 (13.6)
Permanent	14 (31.8)
pulmonary vein isolation, *n* (%)	4 (9.1)
transitory ischemic attack, *n* (%)	3 (6.8)
stroke, *n* (%)	7 (15.9)
coronary artery disease, *n* (%)	25 (56.8)
myocardial infarction, *n* (%)	10 (22.7)
heart failure, *n* (%)	10 (22.7)
peripheral vascular disease, *n* (%)	4 (9.1)
chronic kidney disease, *n* (%)	18 (40.9)
chronic liver disease, *n* (%)	3 (6.8)
sleep apnea, *n* (%)	4 (9.1)
prior bleeding, *n* (%)	34 (77.3)
CHA_2_DS_2_-VASc score (IQR)	4 (3–5)
HAS-BLED score (IQR)	4 (3–5)
**Postinterventional antithrombotic regimen**	
dual antiplatelet therapy for 6 months, *n* (%)	44 (100.0)
**Reasons for rehospitalization/severe clinical events during mid-term follow-up**	
myocardial infarction, *n* (%)	1 (2.3)
heart failure, *n* (%)	6 (13.6)
arrhythmia, *n* (%)	2 (4.5)
vascular problem, *n* (%)	2 (4.5)
gastrointestinal bleeding, *n* (%)	8 (18.2)
renal failure, *n* (%)	1 (2.3)
infectious disease, *n* (%)	2 (4.5)
orthopedic, *n* (%)	2 (4.5)
**Bleeding during mid-term follow-up, BARC-Score**	
1, *n* (%)	1 (2.3)
2, *n* (%)	5 (11.4)
3a, *n* (%)	2 (4.5)
≥3b, *n* (%)	0 (0)

The values are given as medians (25th and 75th percentiles) or total numbers (percentage); BARC = Bleeding Academic Research Consortium, BMI = body mass index, CHA_2_DS_2_-VASc = congestive heart failure, hypertension, age ≥ 75 years, diabetes mellitus, prior stroke or TIA or thromboembolism, vascular disease, age 65–74 years, sex category (i.e., female sex), dL = deciliter, HAS-BLED = hypertension, abnormal renal or liver function, prior stroke, prior major bleeding or predisposition to bleeding, labile INR, elderly, i.e., age > 65 years, prior alcohol, drug, or medication usage predisposing to bleeding, IQR = interquartile range, LVEF = left ventricular ejection fraction, kg = kilogram, m = meter, mg = milligram, min = minute, mL = milliliter, pg = picogram, y = years.

**Table 2:** Changes of patients’ characteristics over mid-term follow-up.

Characteristics	T1	T2	*p* Value
**Smoking status**			
never, *n* (%)	37 (84.1)	37 (84.1)	1.000
>1 year former, *n* (%)	1 (2.3)	1 (2.3)	1.000
current, *n* (%)	6 (13.6)	6 (13.6)	1.000
**Pharmacotherapy**			
beta blockers, *n* (%)	17 (38.6)	15 (34.1)	0.825
calcium channel blockers, *n* (%)	9 (20.5)	7 (15.9)	0.783
combined therapy, *n* (%)	14 (31.8)	15 (34.1)	1.000
statines, *n* (%)	27 (61.4)	28 (63.6)	1.000
statin and ezetimibe, *n* (%)	1 (2.3)	1 (2.3)	1.000
other lipid-lowering drugs, *n* (%)	1 (2.3)	1 (2.3)	1.000
**Anticoagulation**			
None	15 (34.1)	43 (97.7)	<0.001
Any	29 (65.9)	1 (2.3)	<0.001
phenprocoumon, *n* (%)	10 (22.7)	0 (0.0)	0.001
dabigatran, *n* (%)	3 (6.8)	0 (0.0)	0.241
rivaroxaban, *n* (%)	3 (6.8)	0 (0.0)	0.241
apixaban, *n* (%)	3 (6.8)	1 (2.3)	0.616
LMWH, *n* (%)	10 (22.7)	0 (0.0)	0.001
**Echocardiographic data**			
LA diameter, mm (IQR)	48.0 (43.7–55.0)	49.0 (44.0–53.0)	0.657
LA surface, cm^2^ (IQR)	24.0 (19.7–28.0)	22.0 (19.0–25.0)	0.010
LA volume, cm^3^ (IQR)	88.5 (70.2–105.3)	83.0 (66.2–100.5)	0.739
**Laboratory values**			
cholesterol, mg/dL (IQR)	143 (124–186)	155 (128–161)	0.370
NT-proBNP, pg/mL (IQR)	975 (455–1429)	981 (488–1852)	0.323
creatinine, mg/dL (IQR)	1.10 (0.96–1.42)	1.24 (1.01–1.71)	0.430
MDRD-GFR, mL/min/1.73 m^2^ (IQR)	65 (43–65)	56 (37–65)	0.140
Hb, g/dl (IQR)	12.4 (10.7–14.5)	10.8 (9.6–12.7)	0.810
anemia < 10 g/dL, *n* (%)	8 (18.2)	8 (18.2)	1.000
average blood glucose, g/dL (IQR)	113 (94–133)	108 (90–112)	0.900
CRP, mg/L (IQR)	5.1 (2.9–11.1)	4.0 (2.9–14.3)	0.560
LDH, U/L (IQR)	198 (176–240)	240 (199–253)	0.930

The values are given as medians (25th and 75th percentiles) or total numbers (percentage); *p* values are based on a dependent Student’s *t*-test or the Fisher’s exact test before and after left atrial appendage closure, *p* < 0.05 indicates statistical significance; cm = centimeter, CRP = C reactive protein, dL = deciliter, g = gram, Hb = hemoglobin, L = liter, LA = left atrial, LDH = lactate dehydrogenase, LMWH = low-molecular-weight heparin MDRD = Modification of Diet in Renal Disease, mg = milligram, min = minute, mL = milliliter, mm = millimeter, NT-proBNP = amino-terminal pro-brain natriuretic peptide, pg = picogram, U = units.

**Table 3:** Metabolite concentrations, standard deviations, and percentage change.

		T1		T2			
Rank	Metabolite	Mean Conc. (µM)	SD	Mean Conc. (µM)	SD	Percentage Change	*p* Value
1	C18:1	0.171	0.051	0.149	0.054	−12.9	**0.005**
2	C2	10.12	5.02	8.41	3.77	−16.9	**0.005**
3	C5	0.206	0.252	0.312	0.481	51.5	**0.013**
4	C14:1	0.119	0.042	0.107	0.043	−10.1	**0.035**
5	C16	0.125	0.034	0.116	0.035	−7.2	0.050
6	C3-DC/C4-OH	0.076	0.043	0.070	0.073	−7.9	0.071
7	C3	0.385	0.161	0.424	0.232	10.1	0.155
8	C4	0.336	0.221	0.360	0.212	7.1	0.337
9	C14:2	0.033	0.021	0.031	0.025	−6.1	0.380
10	C18	0.054	0.024	0.052	0.012	−3.7	0.422
11	C0	36.93	11.42	35.15	10.15	−4.8	0.430
12	C18:2	0.042	0.014	0.041	0.023	−2.4	0.430
13	C10	0.231	0.114	0.220	0.091	−4.8	0.493

*p* values are based on a dependent Student’s *t*-test before and after left atrial appendage closure; *p* < 0.05 indicates statistical significance; µM = micromolar, SD = standard deviation.

**Table 4:** Data adjustment based on a linear mixed model (regression analysis).

Rank	Metabolite	FDR	Beta	Standard Error	*t* Value	*p* Value
1	C18:1	0.0351	−0.236	0.080	−2.961	0.0050
2	C2	0.0351	−0.254	0.087	2.930	0.0054
3	C5	0.0549	0.456	0.175	2.602	0.0127
4	C14:1	0.1130	−0.189	0.087	−2.180	0.0348
5	C16	0.1288	−0.121	0.060	−2.021	0.0495
6	C3-DC/C4-OH	0.1534	−0.186	0.100	−1.853	0.0708
7	C3	0.2886	0.123	0.085	1.446	0.1554
8	C4	0.4663	0.092	0.095	0.971	0.3368
9	C14:2	0.4663	−0.102	0.115	−0.887	0.3801
10	C18	0.4663	−0.056	0.069	−0.810	0.4222
11	C0	0.4663	−0.061	0.077	−0.797	0.4297
12	C18:2	0.4663	−0.071	0.089	−0.796	0.4304
13	C10	0.4932	−0.074	0.107	−0.691	0.4932

*p* values are based on a linear mixed-effect model, *p* < 0.05 indicates statistical significance; FDR = false discovery rate.

## Supplementary Materials

Supplementary materials can be found at http://www.mdpi.com/1422-0067/19/2/500/s1.

## Abbreviations

AF	atrial fibrillation
ANOVA	analysis of variance
BMI	body mass index
CAC	carnitine/acylcarnitine carrier protein
CHA_2_DS_2_-VASc	congestive heart failure, hypertension, age ≥ 75 years, diabetes mellitus, prior stroke or TIA or thromboembolism, vascular disease, age 65–74 years, sex category (i.e., female sex)
CPT	carnitine palmitoytransferase
CRP	C reactive protein
CT	computed tomography
EDTA	ethylenediaminetetra-acetic acid
ESI–LC–MS/MS	electrospray ionization–liquid chromatography– mass spectrometry
FDR	false discovery rate
HAS-BLED	hypertension, abnormal renal or liver function, prior stroke, prior major bleeding or predisposition to bleeding, labile INR, elderly, i.e., age > 65 years, prior alcohol, drug, or medication usage predisposing to bleeding
HCA	hierarchical cluster analysis
INR	International Normalized Ratio
LAA	left atrial appendage
LAAC	left atrial appendage
LABEL	Left Atrial Appendage Occlusion and Biomarker Evaluation
LC	long chain
LDH	lactate dehydrogenase
LOD	limit of detection
LV	left ventricular
LVEF	left ventricular ejection fraction
MC	medium chain
MC	medium chain
MDRD	Modification of Diet in Renal Disease
NT-proBNP	amino-terminal pro-brain natriuretic peptide
OAC	oral anticoagulation
PCA	principal component analysis
PLS-DA	partial least squares discrimination analysis
SC	short chain
TCA	tricarboxylic acid
