# Supplementary material for: Interventional Left Atrial Appendage Closure Affects the Metabolism of Acylcarnitines
Source: Int J Mol Sci. 2018 Feb 7;19(2):500. doi: 10.3390/ijms19020500 (PMC5855722; doi:10.3390/ijms19020500)
Supplement: Supplementary file 1 [file ijms-19-00500-s001.pdf]

**SUPPLEMENTAL MATERIAL**

**Table S1 Subgroup analyses for left ventricular ejection fraction**

| | | **T1** | | | | **T2** | | | | | |
|---|---|---|---|---|---|---|---|---|---|---|---|
| | | **Normal LVEF (N=34)** | | **Reduced LVEF (N=10)** | | **Normal LVEF (N=34)** | | **Reduced LVEF (N=10)** | | **rANOVA** | |
| **Rank** | **Metabolite** | **Mean conc. [µM]** | **SD** | **Mean conc. [µM]** | **SD** | **Mean conc. [µM]** | **SD** | **Mean conc. [µM]** | **SD** | **FDR** | **p-Value** |
| 1 | C18 | 0.06 | 0.02 | 0.05 | 0.01 | 0.05 | 0.01 | 0.05 | 0.02 | 0.2275 | **0.0175** |
| 2 | C3 | 0.38 | 0.16 | 0.39 | 0.18 | 0.43 | 0.21 | 0.41 | 0.16 | 0.2672 | **0.0411** |
| 3 | C18:1 | 0.17 | 0.05 | 0.18 | 0.06 | 0.15 | 0.05 | 0.15 | 0.05 | 0.4169 | 0.0962 |
| 4 | C16 | 0.13 | 0.03 | 0.12 | 0.03 | 0.12 | 0.03 | 0.11 | 0.03 | 0.5551 | 0.1708 |
| 5 | C5 | 0.20 | 0.27 | 0.22 | 0.20 | 0.24 | 0.28 | 0.57 | 0.80 | 0.6065 | 0.2835 |
| 6 | C3.DC.-C4.OH | 0.07 | 0.03 | 0.10 | 0.06 | 0.06 | 0.03 | 0.09 | 0.08 | 0.6065 | 0.3095 |
| 7 | C14:1 | 0.11 | 0.03 | 0.14 | 0.06 | 0.11 | 0.05 | 0.11 | 0.04 | 0.6065 | 0.3266 |
| 8 | C2 | 9.21 | 3.78 | 13.21 | 7.07 | 8.49 | 3.92 | 8.10 | 3.20 | 0.6796 | 0.4182 |
| 9 | C4 | 0.30 | 0.17 | 0.46 | 0.22 | 0.34 | 0.21 | 0.43 | 0.19 | 0.693 | 0.5392 |
| 10 | C18:2 | 0.04 | 0.01 | 0.05 | 0.02 | 0.04 | 0.02 | 0.04 | 0.02 | 0.693 | 0.5789 |
| 11 | C0 | 36.69 | 10.88 | 37.78 | 13.04 | 35.89 | 9.71 | 32.64 | 11.19 | 0.693 | 0.6046 |
| 12 | C10 | 0.22 | 0.07 | 0.28 | 0.18 | 0.22 | 0.09 | 0.22 | 0.10 | 0.693 | 0.6538 |
| 13 | C14:2 | 0.03 | 0.01 | 0.04 | 0.02 | 0.03 | 0.02 | 0.03 | 0.01 | 0.693 | 0.693 |

conc. = concentration; FDR = false discovery rate; LVEF = left ventricular ejection fraction; µM = micromolar; SD = standard deviation

**Table S2 Subgroup analyses for renal function**

| | | T1 | | | | T2 | | | | | |
|---|---|---|---|---|---|---|---|---|---|---|---|
| | | **Creatinine > 1.2 (N=14)** | | **Creatinine < 1.2 (N=30)** | | **Creatinine > 1.2 (N=14)** | | **Creatinine < 1.2 (N=30)** | | | |
| **Rank** | **Metabolite** | **Mean conc. [μM]** | **SD** | **Mean conc. [μM]** | **SD** | **Mean conc. [μM]** | **SD** | **Mean conc. [μM]** | **SD** | **FDR** | **p-Value** |
| 1 | C18:1 | 0.19 | 0.06 | 0.16 | 0.04 | 0.16 | 0.06 | 0.14 | 0.05 | 0.1989 | **0.019** |
| 2 | C14:1 | 0.13 | 0.05 | 0.11 | 0.04 | 0.12 | 0.05 | 0.10 | 0.04 | 0.1989 | **0.0306** |
| 3 | C16 | 0.14 | 0.04 | 0.12 | 0.03 | 0.12 | 0.03 | 0.11 | 0.03 | 0.2076 | **0.0479** |
| 4 | C5 | 0.34 | 0.35 | 0.15 | 0.16 | 0.52 | 0.72 | 0.22 | 0.25 | 0.4858 | 0.1498 |
| 5 | C3 | 0.46 | 0.18 | 0.35 | 0.14 | 0.48 | 0.21 | 0.40 | 0.18 | 0.4858 | 0.2317 |
| 6 | C2 | 13.95 | 6.81 | 8.34 | 2.30 | 9.94 | 4.41 | 7.68 | 3.19 | 0.4858 | 0.2946 |
| 7 | C18 | 0.06 | 0.02 | 0.05 | 0.01 | 0.05 | 0.02 | 0.05 | 0.01 | 0.4858 | 0.3026 |
| 8 | C10 | 0.27 | 0.16 | 0.21 | 0.07 | 0.26 | 0.09 | 0.21 | 0.08 | 0.4858 | 0.3206 |
| 9 | C14:2 | 0.04 | 0.02 | 0.03 | 0.01 | 0.04 | 0.02 | 0.03 | 0.01 | 0.4858 | 0.3363 |
| 10 | C18:2 | 0.05 | 0.02 | 0.04 | 0.01 | 0.04 | 0.02 | 0.04 | 0.02 | 0.5025 | 0.3865 |
| 11 | C3.DC-C4.OH | 0.09 | 0.04 | 0.07 | 0.04 | 0.07 | 0.03 | 0.07 | 0.06 | 0.5282 | 0.4469 |
| 12 | C4 | 0.50 | 0.18 | 0.26 | 0.15 | 0.48 | 0.22 | 0.30 | 0.18 | 0.6433 | 0.5938 |
| 13 | C0 | 41.99 | 13.97 | 34.58 | 9.10 | 34.93 | 11.38 | 35.25 | 9.52 | 0.7167 | 0.7167 |

conc. = concentration, FDR = false discovery rate, μM = micromolar, SD = standard deviation

**Table S3 Subgroup analyses for NT-proBNP levels**

| | | T1 | | | | T2 | | | | | |
|---|---|---|---|---|---|---|---|---|---|---|---|
| | | proBNP > 1038 (N=21) | | proBNP < 1038 (N=23) | | proBNP > 1038 (N=21) | | proBNP < 1038 (N=23) | | rANOVA | |
| **Rank** | **Metabolite** | **Mean conc. [μM]** | **SD** | **Mean conc. [μM]** | **SD** | **Mean conc. [μM]** | **SD** | **Mean conc. [μM]** | **SD** | **FDR** | **p-Value** |
| 1 | C5 | 0.18 | 0.19 | 0.23 | 0.30 | 0.34 | 0.61 | 0.29 | 0.31 | 0.1677 | **0.0129** |
| 2 | C16 | 0.13 | 0.03 | 0.12 | 0.03 | 0.12 | 0.03 | 0.11 | 0.03 | 0.2431 | **0.0374** |
| 3 | C18 | 0.05 | 0.02 | 0.05 | 0.01 | 0.05 | 0.02 | 0.05 | 0.01 | 0.2974 | 0.0788 |
| 4 | C18:1 | 0.17 | 0.04 | 0.17 | 0.06 | 0.16 | 0.05 | 0.14 | 0.05 | 0.2974 | 0.0915 |
| 5 | C10 | 0.26 | 0.14 | 0.20 | 0.07 | 0.25 | 0.10 | 0.20 | 0.07 | 0.3948 | 0.1614 |
| 6 | C18:2 | 0.04 | 0.02 | 0.04 | 0.01 | 0.04 | 0.02 | 0.04 | 0.02 | 0.3948 | 0.2071 |
| 7 | C2 | 9.85 | 5.44 | 10.37 | 4.60 | 8.67 | 3.73 | 8.16 | 3.79 | 0.3948 | 0.2359 |
| 8 | C14:1 | 0.12 | 0.05 | 0.11 | 0.04 | 0.12 | 0.05 | 0.10 | 0.04 | 0.3948 | 0.2447 |
| 9 | C14:2 | 0.04 | 0.02 | 0.03 | 0.01 | 0.03 | 0.02 | 0.03 | 0.01 | 0.3948 | 0.2733 |
| 10 | C4 | 0.33 | 0.16 | 0.35 | 0.22 | 0.38 | 0.22 | 0.34 | 0.20 | 0.4337 | 0.3446 |
| 11 | C3.DC-C4.OH | 0.08 | 0.05 | 0.07 | 0.03 | 0.08 | 0.06 | 0.07 | 0.03 | 0.4337 | 0.367 |
| 12 | C3 | 0.35 | 0.14 | 0.42 | 0.18 | 0.43 | 0.21 | 0.42 | 0.19 | 0.4923 | 0.4544 |
| 13 | C0 | 33.27 | 10.68 | 40.28 | 11.04 | 33.50 | 10.42 | 36.65 | 9.67 | 0.59 | 0.59 |

BNP = brain natriuretic peptide, conc. = concentration, FDR = false discovery rate, μM = micromolar, SD = standard deviation

**Table S4 Subgroup analyses for diabetes mellitus**

| | | T1 | | | | T2 | | | | | |
|---|---|---|---|---|---|---|---|---|---|---|---|
| | | Diabetes (N=16) | | No Diabetes (N=28) | | Diabetes (N=16) | | No Diabetes (N=28) | | | |
| **Rank** | **Metabolite** | **Mean conc. [μM]** | **SD** | **Mean conc. [μM]** | **SD** | **Mean conc. [μM]** | **SD** | **Mean conc. [μM]** | **SD** | **FDR** | **p-Value** |
| 1 | C0 | 37.55 | 14.55 | 36.58 | 9.15 | 36.58 | 11.28 | 34.41 | 9.43 | 0.0351 | **0.0027** |
| 2 | C18:1 | 0.17 | 0.05 | 0.17 | 0.05 | 0.16 | 0.06 | 0.14 | 0.05 | 0.1242 | **0.0325** |
| 3 | C18:2 | 0.04 | 0.01 | 0.04 | 0.01 | 0.04 | 0.02 | 0.04 | 0.01 | 0.1242 | **0.0368** |
| 4 | C16 | 0.13 | 0.04 | 0.12 | 0.03 | 0.12 | 0.03 | 0.11 | 0.03 | 0.1242 | **0.0382** |
| 5 | C2 | 11.72 | 6.31 | 9.21 | 3.83 | 10.03 | 4.63 | 7.56 | 2.90 | 0.1685 | 0.0648 |
| 6 | C5 | 0.31 | 0.35 | 0.15 | 0.14 | 0.41 | 0.40 | 0.26 | 0.51 | 0.4621 | 0.2398 |
| 7 | C3.DC-C4.OH | 0.08 | 0.04 | 0.07 | 0.05 | 0.08 | 0.04 | 0.07 | 0.06 | 0.4621 | 0.2488 |
| 8 | C14:1 | 0.12 | 0.04 | 0.12 | 0.04 | 0.12 | 0.05 | 0.10 | 0.04 | 0.4815 | 0.2963 |
| 9 | C3 | 0.45 | 0.20 | 0.35 | 0.13 | 0.54 | 0.24 | 0.36 | 0.14 | 0.5357 | 0.3971 |
| 10 | C18 | 0.05 | 0.02 | 0.05 | 0.01 | 0.05 | 0.01 | 0.05 | 0.01 | 0.5357 | 0.4428 |
| 11 | C4 | 0.41 | 0.20 | 0.30 | 0.18 | 0.47 | 0.25 | 0.30 | 0.16 | 0.5357 | 0.5107 |
| 12 | C14:2 | 0.03 | 0.02 | 0.03 | 0.01 | 0.04 | 0.02 | 0.03 | 0.01 | 0.5357 | 0.5225 |
| 13 | C10 | 0.24 | 0.16 | 0.23 | 0.07 | 0.24 | 0.09 | 0.22 | 0.09 | 0.5357 | 0.5357 |

conc. = concentration, FDR = false discovery rate, μM = micromolar, SD = standard deviation
